# TNF-α augments CXCL10/CXCR3 axis activity to induce Epithelial-Mesenchymal Transition in colon cancer cell

**DOI:** 10.7150/ijbs.61350

**Published:** 2021-06-26

**Authors:** Zhengcheng Wang, Xiang Ao, Zhilin Shen, Luoquan Ao, Xiaofeng Wu, Chengxiu Pu, Wei Guo, Wei Xing, Min He, Hongfeng Yuan, Jianhua Yu, Ling Li, Xiang Xu

**Affiliations:** 1 Department of Human Anatomy and Histology and Embryology, School of Basic Medical Sciences, Qingdao University, Qingdao 266000, China.; 2 Department of Stem Cell & Regenerative Medicine, State Key Laboratory of Trauma, Burn and Combined Injury, Daping Hospital, Army Medical University, Chongqing 400042, China.; 3 Department of Ophthalmology, Daping Hospital, Army Medical University, Chongqing 400042, China.

**Keywords:** CC, TNF-α, CXCL10/CXCR3, migration, invasion, EMT

## Abstract

Chronic inflammation-induced metastases have long been regarded as one of the significant obstacles in treating cancer. Tumor necrosis factor-α (TNF-α), a main inflammation mediator within tumor microenvironment, affects tumor development by inducing multiple chemokines to establish a complex network. Recent reports have revealed that CXCL10/CXCR3 axis affects cancer cells invasiveness and metastases, and Epithelial-mesenchymal transition (EMT) is the main reason for frequent proliferation and distant organ metastases of colon cancer (CC) cells, However, it is unclear whether TNF-α- mediated chronic inflammation can synergically enhance EMT-mediated CC metastasis through promoting chemokine expression. According to this study, TNF-α activated the PI3K/Akt and p38 MAPK parallel signal transduction pathways, then stimulate downstream NF-κB pathway p65 into the nucleus to activate CXCL10 transcription. CXCL10 enhanced the metastases of CC-cells by triggering small GTPases such as RhoA and cdc42. Furthermore, overexpression of CXCL10 significantly enhanced tumorigenicity and mobility of CC cells *in vivo*. We further clarified that CXCL10 activated the PI3K/Akt pathway through CXCR3, resulting in suppression of GSK-3β phosphorylation and leading to upregulation of Snail expression, thereby regulating EMT in CC cells. These outcomes lay the foundation for finding new targets to inhibit CC metastases.

## Introduction

Colon cancer (CC) accounts for one of the frequently occurring gastrointestinal malignant tumors, which ranks the 3^rd^ and 4^th^ places in terms of morbidity and mortality of all cancers, respectively 1, 2. The high mortality rate in CC has been attributed to the nuanced clinical variation in patients with CC and a shortage of responsive early diagnostic biomarkers and successful treatment methods. This is partly attributable to the recurrent proliferation of CC cells and remote organ metastases. Epithelial-mesenchymal transition (EMT) stands for the main link of tumor metastasis and invasion 3. With progress in cancer functional genomics, people have an increasingly comprehensive understanding of the incidence and progress of CC. Nevertheless, the molecular mechanism of EMT-mediated CC metastasis is still unclear. Abundant studies have shown that EMT is a crucial factor in invasiveness and metastases of breast tumor, CC, pancreatic cancer, prostate, and other solid cancers 4-8. EMT refers to the progression course where epithelial cells secure the mesenchymal phenotype through diverse complex specific events. During EMT, tumor cells gain the fibroblast-like phenotype from the epithelial phenotype, which leads to polarity loss as well as epithelial cell adhesion, enhancement of their activity and invasiveness, thus triggering the invasiveness and metastases of malignant cancers 9, 10. During EMT feature's progression, epithelial-marker's decreases, while mesenchymal-markers express highly 11-13. Many growth chemokines and inflammatory factors regulate EMT during tumor metastasis 14. Chemokines contribute significantly to the associations between tumor cells and the surrounding mesenchymal cells, according to recent research. Chemokines, as key signaling molecules in the tumor microenvironment, induce the relocation of tumor cells that expresses chemokine-receptors inwards the signal-gradient where chemokine concentrations are increased, this is one of the important reasons of progression for the malignant tumors, for example, tumor invasiveness and target organ metastases 15-18. However, the role of paracrine and/or autocrine chemokine signals in EMT-mediated CC metastases remain unclear.

CXCL10 is a proinflammatory cytokine secreted by immune cells 19. CXCL10 inhibits tumorigenesis by interacting with CXCR3 receptors through paracrine and/or autocrine signaling, mediating immune cell chemotaxis, differentiation, and activation 20. CXCL10, on the other hand, has been shown to stimulate tumor growth and metastases by an autocrine axis 21, 22 and recently, it was discovered to be linked to a bad prognosis in patients with CC 23, 24. Furthermore, the expression of every chemokine's homologous receptor determines the chemokine's host cells specificity. CXCR3 is a G protein-coupled receptor (GPR) that contains 7 transmembrane domains, which can generate secondary signals and activate multiple downstream pathways through specific binding with the ligands CXCL9-11 25; subsequently mediating tumor formation, and the proliferation, apoptosis, angiogenesis, invasiveness and metastases of cancer cells, thereby leading to the progression of many diseases 23, 26. Clinical data analysis showed that CXCR3 had elevated expression in melanoma, colon, ovarian and basal cell carcinoma 27-33. Furthermore, CXCR3 level within breast cancer (BC) cells aided bone metastasis occurrence 34, and CXCR3 antagonists have been used to hinder lung metastases in metastatic breast tumor 35. From what has been discussed above, a large amount of data clearly suggests the relationships between CXCR3/CXCL10 co-expression and progression of early tumor metastases and an increase in metastatic potential. However, CXCL10/CXCR3 signal transduction conduit mechanism underlying poor prognosis and CC metastasis remains to be explored.

In the tumor microenvironment (TME), complex networks comprising chemokines together with the corresponding receptors can promote cancer development via damage to and escape of immune surveillance, thus affecting the occurrence and development of tumors 36, 37. However, notably, the acquisition and maintenance of this promotive effect on the metastatic tumor potential requires continuous stimulatory signals from the matrix microenvironment, among them, pathogenic stimuli, growth factors, and inflammatory cytokines account for the primary causes 15. Tumor necrosis factor-α (TNF-α) represents the critical mediating factor for tumor-related inflammation in the tumor area, and it can cause a number of chemokines to be generated 38-45, chronic low-dose TNF-α can even cause dismal prognostic outcome, hormone tolerance, and cachexia, and has been shown to induce tumor growth and metastases 46-49, but it is unclear whether TNF-α-mediated chronic inflammation can synergistically enhance EMT-mediated CC metastases by inducing the expression of chemokines. In the current project, it was concluded that TNF-α increased CXCL10 expression via the p38 MAPK, NF-κB and PI3K/Akt pathways. Additionally, CXCL10 caused EMT in CC cells through the CXCR3-regulated PI3K/Akt/GSK-3β/Snail signal transduction pathway. On the other hand, CXCL10 enhanced the migration and invasiveness of CC cells via activating small GTPases such as RhoA and cdc42. In summary, our study revealed a new role of the CXCL10/CXCR3 axis activated by TNF-α in regulating EMT and CC invasiveness and metastases via PI3K/Akt/GSK-3β/Snail signal transduction pathway and this study can be a helpful for the researchers exploring recurrent and metastatic treatment of CC.

## Materials and methods

### Cell culture and reagents

CC cell lines (SW480/SW620) were purchased from the American Type Culture Collection (ATCC, USA), cultivated within L15 media that contained 10% FBS (VivaCell, Shanghai, China) and incubated in the ventilated environment under 37 °C and 95% air and 5% CO_2_ conditions. Recombinant human TNF-α was obtained from Sino Biological, and recombinant human CXCL10 was purchased from Peprotech. At the final concentrations listed, the following basic pharmacological inhibitors were used: SB203580 (the inhibitor of p38 MAPK, Selleck), LY294002 (the inhibitor of PI3K, Selleck), MK-2206 2HCI (the inhibitor of Akt, Selleck), BAY11-7082 (the inhibitor of NF-κB, Selleck) and CHIR-99021 (the inhibitor of GSK-3β, MCE). All the cellular studies were carried out under specified and standard conditions.

### ELISA

The concentration of CXCL10 secreted by TNF-α induced CC cells was determined by ELISA assay. A CXCL10 ELISA kit was purchased from Boster (Wuhan, China), SW480/SW620 cells were cultivated into the 6-well plate, exposed to TNF-α stimulation at the specified concentration and time point, and the cell supernatant was collected, the concentration of CXCL10 was detected in line with specific detection kit protocols step by step.

### Flow cytometry

This study performed flow cytometry to detect CXCR3 expression on CC cell membrane. We cultured SW480/SW620 cells within the six-well plates and exposed them to TNF-α for a period of 24 h. After cell digestion and collection, cells were resuspended with antibody labeling solution and count (1×10^6^ cells/100 μl), 100 μl sample blocking solution was added into every group, followed by 30 min of incubation under 4 °C, in addition, all groups were added with Isotype control (Mouse IgG1 PE-labeled Antibody/R&D Systems) and detection antibody (Human CXCR3 PE-labeled Antibody/R&D Systems), followed by 30 min of incubation under 4 °C in dark. 2 ml antibody labeling solution was added, followed by centrifugation and removal of supernatants. Later, PBS was used to resuspend cells and flow cytometry was conducted to examine cells.

### RT-qPCR

This study utilized SteadyPure Universal RNA Extraction Kit (Accurate Biology) to extract total RNA, and cDNA was synthesized by Evo M-MLV Mix Kit with gDNA Clean for qPCR (Accurate Biology). This study also performed qPCR assay by the use of SYBR® Green Premix Pro Taq HS qPCR Kit (Accurate Biology). All primer sequences adopted in PCR were shown below. CXCL10: (F) 5′-AGCAAGGAAAGGTCTAAAAGATCTCC-3′ and (R) 5′-GGCTTGACATATACTCCATGTAGGG-3′, CXCR3: (F) 5′-GCTCTGAGGACTGCACCATTG-3′ and (R) 5′-TGAAGTTTTAGTTTCCAAATGAGAAGGG-3′, E-cadherin:(F) 5′-TACACTGCCCAGGAGCCAGA-3′ and (R) 5′-TGGCACCAGTGTCCGGATTA-3′, N-cadherin: (F) 5′-CGAATGGATGAAAGACCCATCC-3′ and (R) 5′-GGAGCCACTGCCTTCATAGTCAA-3′, Fibronectin: (F) 5′-ACAAACACTAATGTTAATTGCCCA-3′ and (R) 5′-GAACTCTAAGCTGGGTCTGCT-3′, Snail: (F) 5′-GACCACTATGCCGCGCTCTT-3′ and (R) 5′-TCGCTGTAGTTAGGCTTCCGATT-3′, Twist: (F) 5′-GGAGTCCGCAGTCTTACGAG-3′ and (R) 5′-TCTGGAGGACCTGGTAGAGG-3′,Vimentin: (F) 5′-CGGGAGAAATTGCAGGAGGA-3′ and (R) 5′-AAGGTCAAGACGTGCCAGAG-3′, Occludin: (F) 5′-TTGCGGCGAGCGGATTG-3′ and (R) 5′-TGGACTTTCAAGAGGCCTGG-3′, ZO-1: (F) 5′-CAACATACAGTGACGCTTCACA-3′ and (R) 5′-CACTATTGACGTTTCCCCACTC-3′, ZEB-1: (F) 5′-TACAGAACCCAACTTGAACGTCACA-3′ and (R) 5′-GATTACACCCAGACTGCGTCACA-3′, Slug: (R) 5′-TTCGGACCCACACATTACCT-3′ and (R) 5′-GCAGTGAGGGCAAGAAAAAG-3′, Cytokeratin: (F) 5′-TCGCCAAGATCCTGAGTGAC-3′ and (R) 5′-CAATTCTTCAGTCCGGCTGGT-3′, Demsoplakin: (F) 5′-AAAGCCATCAGTGTCCCTCG-3′ and (R) 5′-ATGTCCATCTCCGCCCTTTG-3′, GAPDH: (F) 5′-TCGACAGTCAGCCGCATCTTC-3′ and (R) 5′-CGCCCAATACGACCACCTCCG-3′. Three separate experiments were used to achieve cDNA amplification and relative expression rates, and expression was quantified by comparative period threshold (Ct) process and formula 2(-∆∆Ct).

### WB assay

WB assay was used to determine the changes of related proteins. In brief, we cultured SW480/SW620 cells into the 6-well plate and exposed them to TNF-α or CXCL10 stimulation for a specified time. Cells were collected and the Column Tissue&Cell Protein Extraction Kit (Epizyme) was employed to extract total protein. Protein concentration was measured by Omni-Easy™ Instant BCA Protein Assay Kit (Epizyme). The PAGE Gel Fast Preparation Kit (Epizyme) was utilized to prepare the gel for electrophoresis; proteins were then transferred to PVDF membranes, followed by blocking using 5% skim milk contained within TBST for a period of 1.5 h before overnight incubation using primary antibodies under 4 °C. Primary antibodies included including anti-N-cadherin, E-cadherin, fibronectin, CXCR3, Vimentin, p38/p-p38, p65/p-p65, PI3K/p-PI3K, Akt/p-Akt, GSK-3β/p-GSK-3β, Snail, Twist, RhoA, cdc42, Slug, ZEB-1, IKKα, IKKβ, IκBα, β-catenin, LaminB and GAPDH rabbit antibodies (1:1200, CST). Then, cells were incubated using HRP-labeled anti-rabbit IgG antibody (1:12000, CST) for 2 h, The Omni-ECL™Enhanced Pico Light Chemiluminescence Kit (Epizyme) was used for protein luminescence detection on a Bio-Rad gel imaging instrument.

### siRNAs and transfection

siGSK-3β, siSnail and siCXCR3 were obtained from Guangzhou RiboBio (Guangzhou, China). By using Lipo8000™ Transfection Reagent (Beyotime), SW480/SW620 cells were transfected with siRNA. The overexpression of CXCL10 plasmid (vCXCL10) and the no-load irradiation plasmid (Vector) were purchased from Biomedicine Biotechnology (Chongqing, China). The plasmids were transfected into SW480/SW620 cells using NDE3000 Nano polymer Transfection Reagent, the amount of transfection reagents and methods and procedures were operated in line with specific protocols, fluorescence observation and WB assays were performed to detect transfection efficiency (overexpression and interference), and the constructed cells were used for subsequent functional experiments, in addition, mice were given subcutaneous injection of SW480/SW620 cells transfected with vCXCL10 or vector.

### Gene Promoter activity assay

The pNFκB-Luc plasmid was purchased from Beyotime, and Shanghai OBiO Technology provided the CXCL10 promoter plasmids. We cultured SW480/SW620 cells into the 6-well plate and used Lipofectamine 2000 to transfect CXCL10 promoter plasmid, Renilla luciferase plasmid and p65 overexpression plasmid into cells. Fluorescence intensity was detected by Dual Luciferase Reporter Gene Assay Kit (Beyotime) as instructed by instruction manual.

### Wound healing assay

We conducted scratch assay *in vitro* for evaluating CC cell migration. To this end, we cultured SW480/SW620 cells into the 6-well plate and exposed them to CXCL10 stimulation, for each well group, draw 3 vertical lines with 10 μl micropipette tip as the wound, the necrotic cells were washed and removed by PBS, and all wells were added with 1 ml serum-free L15 medium, cell migration rate was detected and measured microscopically for 48 hours.

### Transwell assay

This study conducted Transwell assay for detecting CC cell invasion. In brief, we cultured SW480/SW620 cells within the 24-well (PC-Transwell/Corning) plates and exposed them to CXCL10 for 48 h, Matrigel was smeared on upper chamber membrane, SW480/SW620 in the upper compartment were cultured within the serum-free medium, while 400 microliter L15 medium that contained 15% FBS was added into the lower compartment. Then, cotton was used to wipe those uninvaded cells, followed by PBS washing, methanol fixation, and crystal violet staining, whereas those invaded cells were observed, and their number was calculated under microscope.

### Colony formation assay

CC cell ability to grow un-anchoring was examined by colony formation assay. Vector and CXCL10 plasmids were transfected into SW480/SW620 cells and cultured in six-well plates for 7 days, change the L15 medium every 2 days, fixed cells with paraformaldehyde, crystal violet staining, and the pictures were captured with the mobile phone to calculate colony numbers.

### Immunofluorescence

Immunofluorescence assay was conducted to detect the expression of EMT-associated proteins in CC. We cultured SW480/SW620 cells within Beyogold™ 35 mm Confocal Dishes (Beyotime) and stimulated them with CXCL10. After rinsing by PBS, cells were subjected to paraformaldehyde fixation, and sealing using immunostaining blocking solution under ambient temperature for 1 h. Meanwhile, we cultured SW480/SW620 cells under 4 °C overnight using E-cad, N-cad, fibronectin, or Vimentin antibody (1:200, CST), rinsed them by PBS twice and cultured them using Alexa Fluor 488/555/594/647 fluorescence secondary antibody (1:1200, CST) for 2 h, DAPI was used to stain nuclei (blue), fluorescence intensity expression of EMT-associated proteins was detected by confocal microscopy, all the reagents required above were from Immunofluorescence Application Solutions Kit (CST).

### Pull down assay

GTP-binding protein activity within CC cells stimulated by CXCL10 was detected by pull down assay. Endogenous expression of GTP-binding cdc42 was detected by the active cdc42 Detection Kit (CST), while that of GTP-binding RhoA was measured by Active Rho Detection Kit (CST). We then cultivated SW480/SW620 cells within the 6-well plate and then exposed them to CXCL10, cells were lysed, and proteins were collected. GST-PAK1-PBD and GST-Rhotekin-RBD were thawed on ice and added to a swirling cup containing glutathione resin. Scroll and wash the resin, then add a sample reduction buffer to elute the sample protein. Finally, the collected proteins were tested by WB assay for the expression of GTP-RhoA and GTP-cdc42.

### F-actin staining assay

We conducted microfilament staining assay for detecting recombination and morphologic alterations of F-actin within CC cells. SW480/SW620 were cultured on six-well plates and stimulated with CXCL10. Immunol Staining Fix Solution (Beyotime) fixed the cells for 15 min, and Actin-Tracker Green-488 (Beyotime) was added into the culture well for dark incubation for 30 min. After rinsing cells by PBS twice, we observed them using the fluorescence microscope.

### *In vivo* tumor metastases assay

We purchased seven to nine weeks old BALB/c nude mice from Biocytogen (Beijing, China). The Institutional Animal Care and Use Committee of Daping Hospital of Army Medical University approved the animal experiment protocols. The animals were classified as 6 groups, with 5 in each group, followed by anesthesia. Each anaesthetized nude mouse's inoculation site was disinfected with 70% alcohol then the mouse was inoculated with 230 μl of one of the cell suspensions. Physiological conditions of mice were observed regularly every day, tumor volumes were recorded at intervals of 3 days. At 21 days later, we sacrificed the animals to collect tumors for Ki-67 immunohistochemical assay, followed by H&E staining of lung tissue, and metastatic lesion number was observed and calculated.

### Statistical analysis

Data were displayed in a form of mean ± SD from 3 independently performed assays except as otherwise noted. Significant differences between two groups were examined by Student's t-test, while those among several groups were examined by one-way ANOVA with Sidak multiple comparison test. These analyses were performed by GraphPad Prism (version 9.0). P<0.05 stood for statistical significance.

## Results

### CXCL10/CXCR3 are highly expressed in human colon cancer (CC)

**Figure 1:**
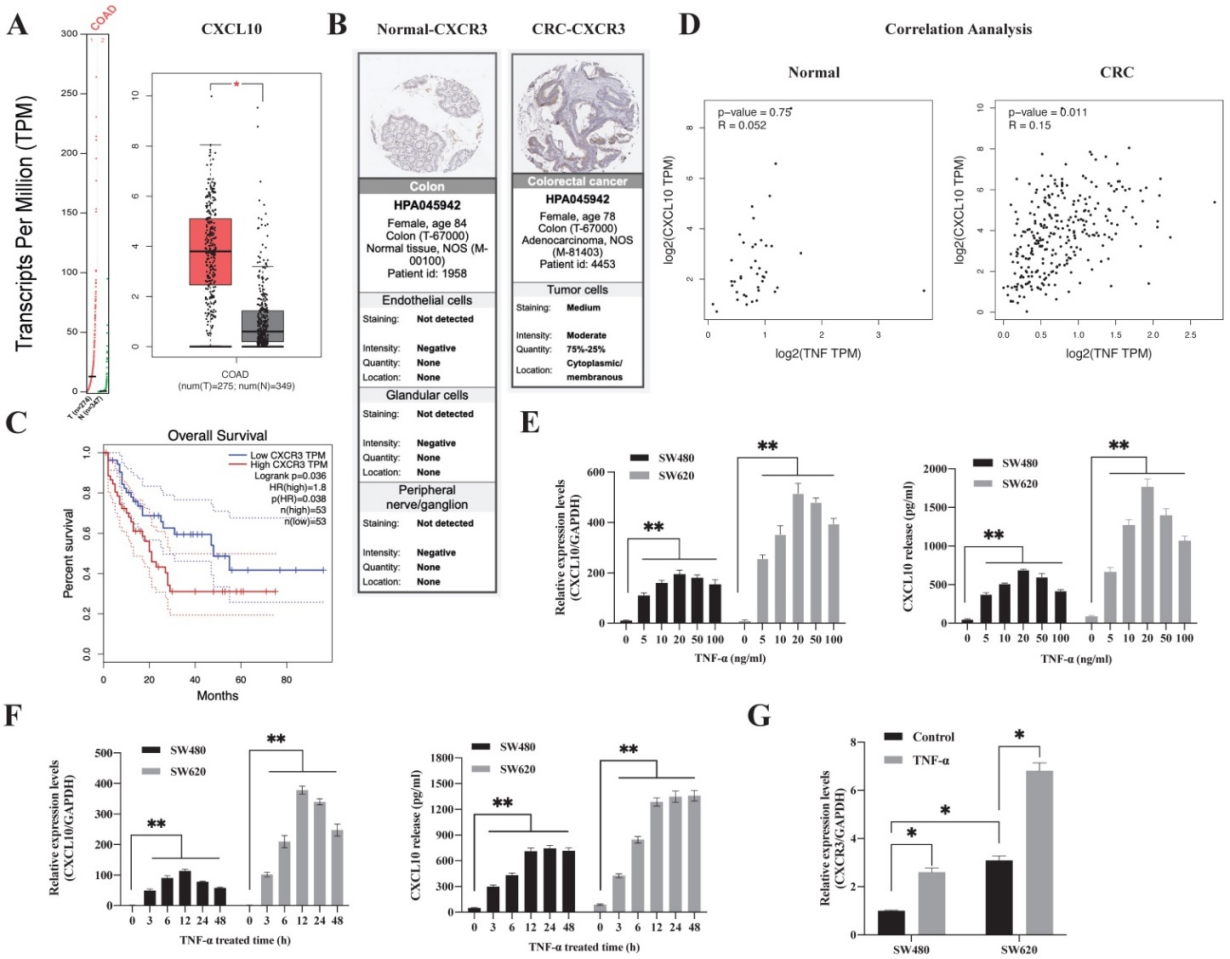
** TNF-α-regulated CXCL10/CXCR3 are highly expressed in CRC. (A)** CXCL10 expression within CRC samples (T) and paired non-carcinoma samples (N). The data are from GEPIA, *p<0.01 versus control.** (B)** CXCR3 levels with normal and CRC tissues. Acquired images from the Human Protein Atlas online database. **(C)** Kaplan-Meier survival curve showing CRC survival curve for patients showing high or low CXCR3 level. **(D)** Association of TNF-α expression with CXCL10 gene levels within CRC and normal samples. Data were obtained from online GEPIA database. **(E)** The TNF-α-induced CXCL10 mRNA and protein levels at specific concentrations in SW480/SW620 cells were measured through ELISA and qPCR. **(F)** The TNF-α-induced CXCL10 mRNA and protein levels at specific time points in SW480/SW620 cells were measured through ELISA and qPCR **(G)** The CXCR3 mRNA expression induced by TNF-α in SW480/SW620 cells was detected by qPCR. Bar charts stand for mean ±SD (n=3). *p< 0.05, **p < 0.01 by Sidak multiple comparison test.

To explore the difference in the CXCL10/CXCR3 biological axis between normal and tumor tissues, we performed GEPIA database analysis, which showed that the CXCL10 gene was significantly overexpressed in CC (Figure 1A). The clinical correlation of CXCR3 receptor expression was evaluated with clinical specimens in the Human Protein Atlas. Figure 1B revealed that, CXCR3 showed high expression within CC tissues and having reduced expression in normal tissues. Following that, we utilized the GEPIA database for investigating the connection of CXCR3 levels with disease prognosis. As revealed by the survival curve, patients showing positive CXCR3 level within CC tissues were associated with reduced survival rate compared with patients showing negative CXCR3 expression (Figure 1C), this suggests that CXCR3 overexpression in CC patients was linked to a shorter survival period. Notably, CXCL10 expression needs continuous stimulatory signals from the tumor microenvironment. TNF-α represents the main regulatory factor for tumor-associated inflammation within TME and plays a vital role in inducing chemokine production and regulating inflammation and immune processes. Based on these observations, we evaluated the degree of association between CXCL10 and TNF-α expression within tumor and normal tissues, we found that CXCL10 and TNF-α expression was positively correlated within CC tissues but not in normal tissues (Figure 1D), suggesting that TNF-α may be related to CXCL10 expression within CC. To sum up, these findings show that TNF-α, CXCL10 and CXCR3 possibly exert an important part in CC development.

### TNF-α regulates CXCL10/CXCR3 levels within CC cells

Subsequently, this study analyzed the time and concentration-dependent impacts of TNF-α - induced CXCL10 in CC. As shown in Figure 1E, TNF-α synchronized the expression of CXCL10 in a concentration-dependent mode. TNF-α (5 ng/ml) was sufficient to induce the transcription of CXCL10, and 20 ng/ml had the strongest induction effect. At concentrations higher than 20 ng/ml, the effect on CXCL10 expression gradually weakened with increasing TNF-α concentration. After 3 h of CXCL10 treatment in SW480 and SW620 cells, CXCL10 mRNA expression was significantly increased, with the highest transcriptional activity of CXCL10 at 12 h (Figure 1F). The protein expression of CXCL10 was significantly augmented following 3 h of TNF-α treatment, peaked at 12 h and stabilized at 24 h (Figure 1F). Importantly, CXCL10 mRNA and protein expression within the metastatic SW620 CC cells were significantly higher than those in SW480 primary CC cells. Moreover, we assessed CXCR3 level within SW480/SW620 cells, as shown in Figure S1A, according to WB assay, CXCR3 expression within the metastatic CC cell line SW620 increased relative to SW480 primary CC cells, the results of flow cytometry were consistent with those of WB analysis (Figure S1C). In addition, TNF-α promoted CXCR3 mRNA expression (Figure 1G). TNF-α's effect on CXCR3 expression was further verified by flow cytometry; as shown in Figure S1D, TNF-α promoted CXCR3 expression within SW480/SW620 cells in a dose-dependent mode. In summary, these outcomes recommend that TNF-α can upregulate CXCL10 and CXCR3 in CC cells.

### TNF-α upregulates CXCL10 level within CC cells mediated via the p38 MAPK, NF-κB and PI3K/Akt signal transduction pathways

**Figure 2:**
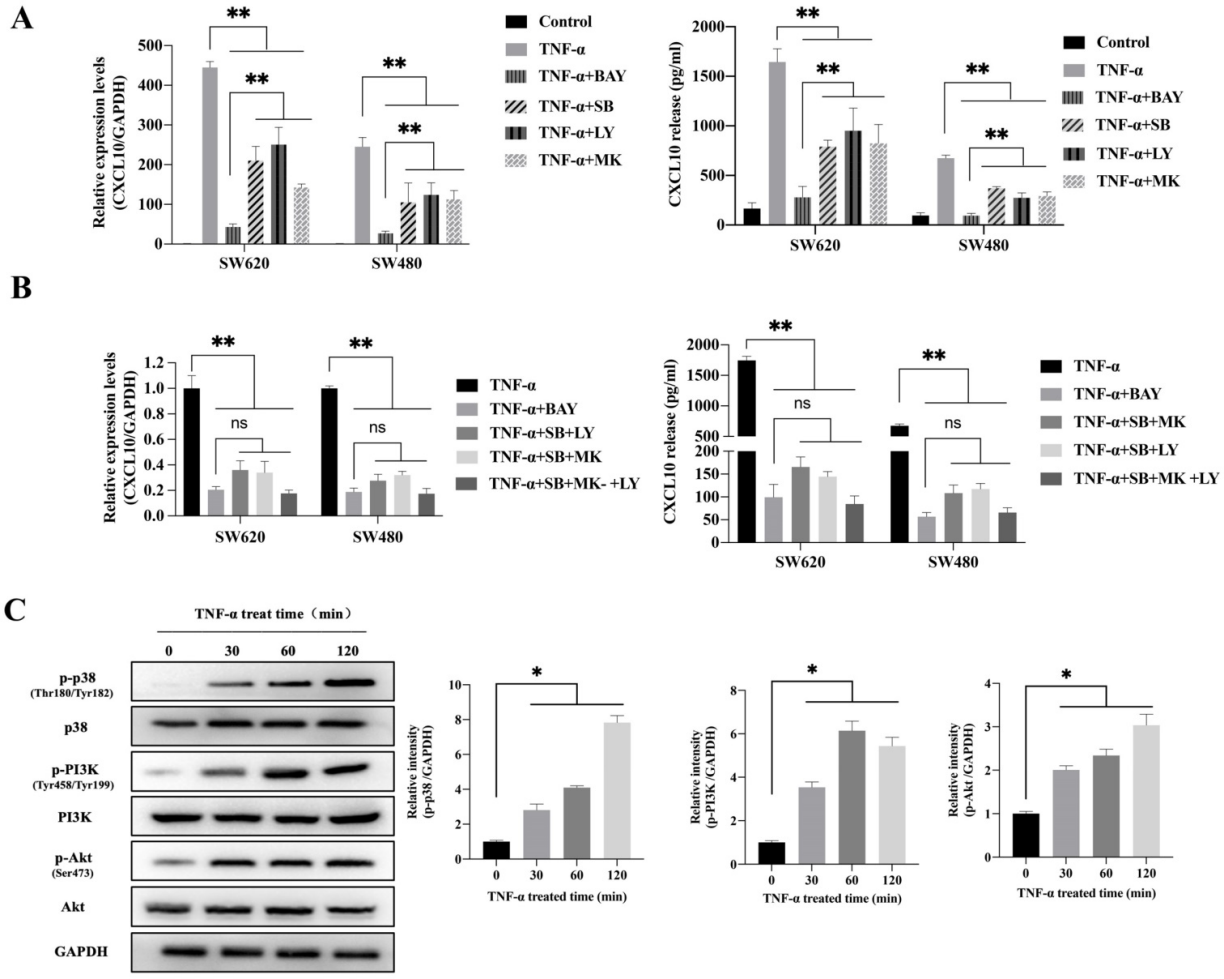

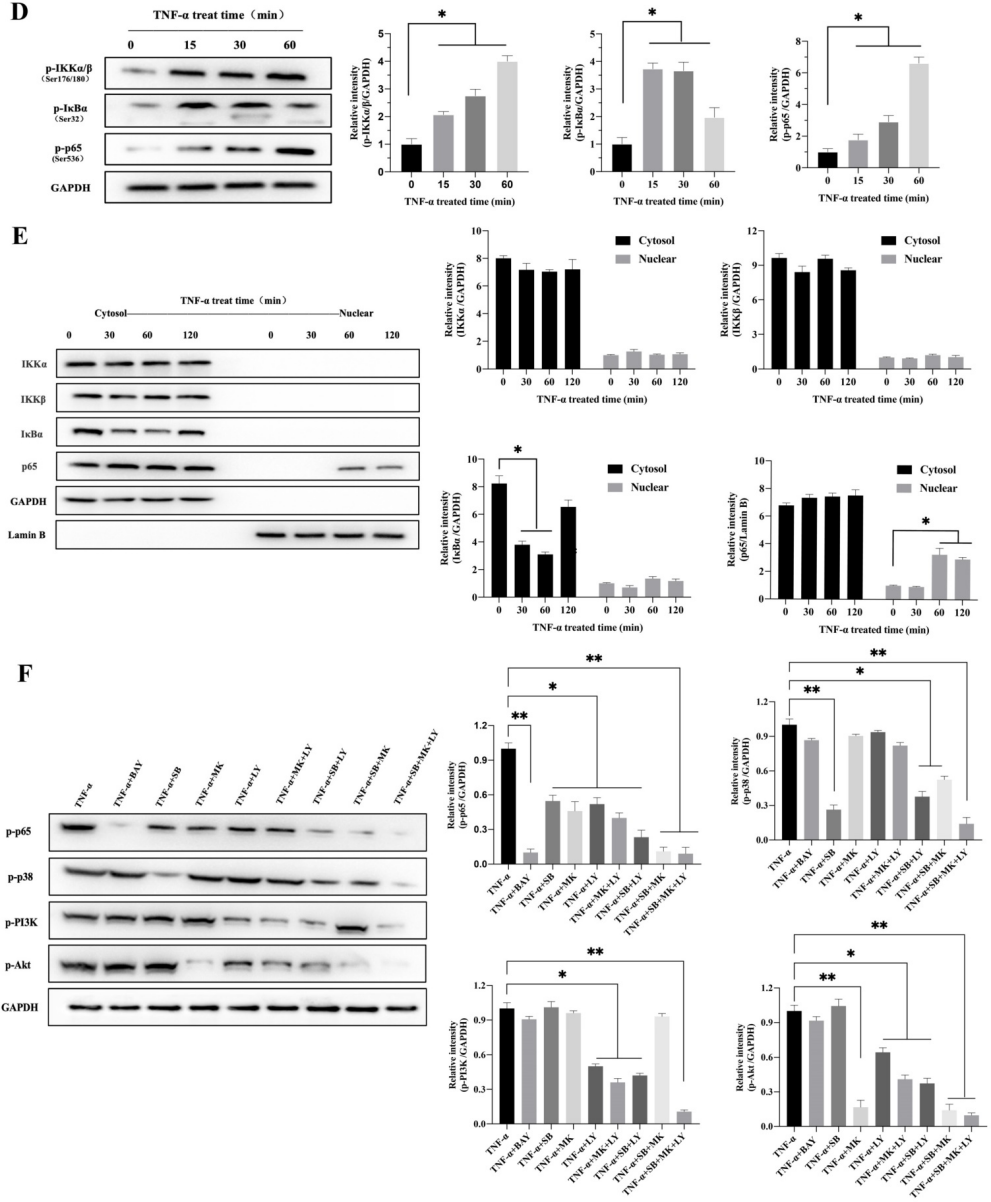
** TNF-α up-regulates CXCL10 level within CC cells by p38 MAPK, NK-κB and PI3K/Akt pathways. (A)** NF-κB pathway inhibitor (BAY11-7082, BAY; targeting NF-κB), a p38 pathway inhibitor (SB203580, SB; targeting p38 MAPK), a PI3K pathway inhibitor (LY294002, LY; targeting PI3K) or an Akt pathway inhibitor (MK-2206 2HCI, MK; targeting Akt) was used to treat SW480/SW620 cells for 30 min before 24 h of 20 ng/ml TNF-α stimulation. At 24 h later, CXCL10 mRNA and protein levels were measured through qPCR and ELISA. **(B)** BAY, SB, LY, or MK was used to treat SW480/SW620 cells for 30 min together or separately before stimulation with 20 ng/ml TNF-α. At 24 h later, CXCL10 mRNA and protein expression was detected through qPCR and ELISA.** (C)** p-p38/p38, p-Akt/Akt and p-PI3K/PI3K protein levels within SW620 cells stimulated by 20 ng/ml TNF-α were detected through WB analysis. **(D)** p-p65, p-IKKα/β and p-IκBα protein levels within SW620 cells stimulated by 20 ng/ml TNF-α were detected through WB analysis. **(E)** IKKα, IKKβ, p65 and IκBα protein levels cytoplasm and nucleus of SW620 cells after TNF-α (20 ng/ml) stimulation were measured through WB analysis. **(F)** BAY, SB, LY, or MK was used to treat SW620 cells for 30 min together or separately before stimulation using TNF-α (20 ng/ml). At 2 h later, p-p65, p-p38, p-PI3K, and p-Akt protein levels were measured through WB assay in SW620 cells. The anti-GAPDH and anti-Lamin B antibody were used as the internal control, and every bar chart stand for mean ±SD (n=3). *p < 0.05, **p < 0.01 by Sidak multiple comparison test.

To explore the possible signaling pathway by which TNF-α induces CXCL10 expression, we assessed the effects of various signaling pathway inhibitors on CXCL10 expression induced by TNF-α. According to Figure 2A, inhibiting NF-κB pathway abolished the TNF-α stimulated upregulation of CXCL10 mRNA expression, while suppressing PI3K/Akt and p38 MAPK pathways only partially reduced the TNF-α stimulated upregulation of CXCL10 mRNA expression. Under the same treatment conditions, similar results were obtained by ELISA to detect the changes in the concentration of CXCL10 in the culture supernatant of tumor cells (Figure 2A). Surprisingly, we also found that treatment with the combination of inhibitors of both PI3K/Akt and p38 MAPK pathways produced same influence as NF-κB pathway inhibition (Figure 2B). The above results suggest that the PI3K/Akt, NF-κB, and p38 MAPK pathways may participate in TNF-α-induced upregulation of CXCL10 expression in CC cells. WB analysis was further used to explore whether TNF-α can activate the p38 MAPK, NF-κB and PI3K/Akt pathways within CC cells. According to our results, TNF-α significantly enhanced components of the PI3K/Akt and p38 MAPK pathway component phosphorylation within SW620 cells (Figure 2C), phosphorylation levels of p38 MAPK, PI3K and Akt increased significantly after TNF-α treatment for 30 min, p38 MAPK phosphorylation increased depending on time within 2 h, PI3K and Akt pathway component phosphorylation peaked after 60 min and tended to stabilize after 120 min. In addition to the above-mentioned components in PI3K/Akt and p38 MAPK signal transduction pathways, IKKα/β, IκB and p65 in the NF-κB signaling pathway also showed significant phosphorylation after TNF-α stimulation, and their phosphorylation levels gradually increased within 60 min (Figure 2D). In addition, p65 nuclear translocation and IκB cytoplasmic degradation were considered to signal NF-κB-mediated transcriptional initiation. To explore NF-κB pathway activation by TNF-α within CC, we verified the nuclear and cytoplasmic p65 and IκB expression after TNF-α treatment. The expression of IκB in the cytoplasm was reduced at 60 minutes and restored, while the expression of p65 in the nucleus was significantly increased at 120 minutes (Figure 2E). This finding indicated that TNF-α could promote NF-κB pathway activation within CC cells and promote p65 translocation to the nuclei. We also compared NF-κB, PI3K/Akt and p38 MAPK signaling pathway activation in SW620/SW480 cells after TNF-α stimulation, as shown in Figure S2A. Under stimulation with the same concentration of TNF-α, the p65, Akt and PI3K phosphorylation within SW620 cells were higher than those in SW480 cells, which may explain why SW620 metastatic CC cells expressed higher levels of CXCL10 than SW480 situ CC cells after stimulation with TNF-α at the same concentration. The above results confirmed that NF-κB, PI3K/Akt, and p38 MAPK signaling pathway inhibitors could affect the TNF-α-induced CXCL10 level within CC cells. Therefore, we evaluated related signal transduction pathway activation in CC cells exposed the above inhibitors was evaluated. As shown in Figure 2F, in addition to specifically inhibiting the activation of corresponding targets, p38 MAPK, PI3K and Akt inhibitors also inhibited p65 phosphorylation when used alone; moreover, the combination of p38 MAPK, PI3K and Akt inhibitors had a stronger inhibitory effect on p65 phosphorylation than any inhibitor used alone. The change in signaling pathway activation under treatment with these inhibitors was similar to CXCL10 expression under treatment with these inhibitors. The above results indicate that the p38 MAPK, NF-κB and PI3K/Akt signal transduction pathways are related to CXCL10 up-regulation in CC cells by TNF-α. Among these pathways, activating NF-κB pathway, especially p65 nuclear translocation, maybe the key signal for the final initiation of CXCL10 transcription, understanding these signaling pathways in CC is essential for the treatment and prevention of CC metastasis.

### TNF-α promotes NF-κB-p65 transcription factor binding to the CXCL10 promoter

**Figure 3:**
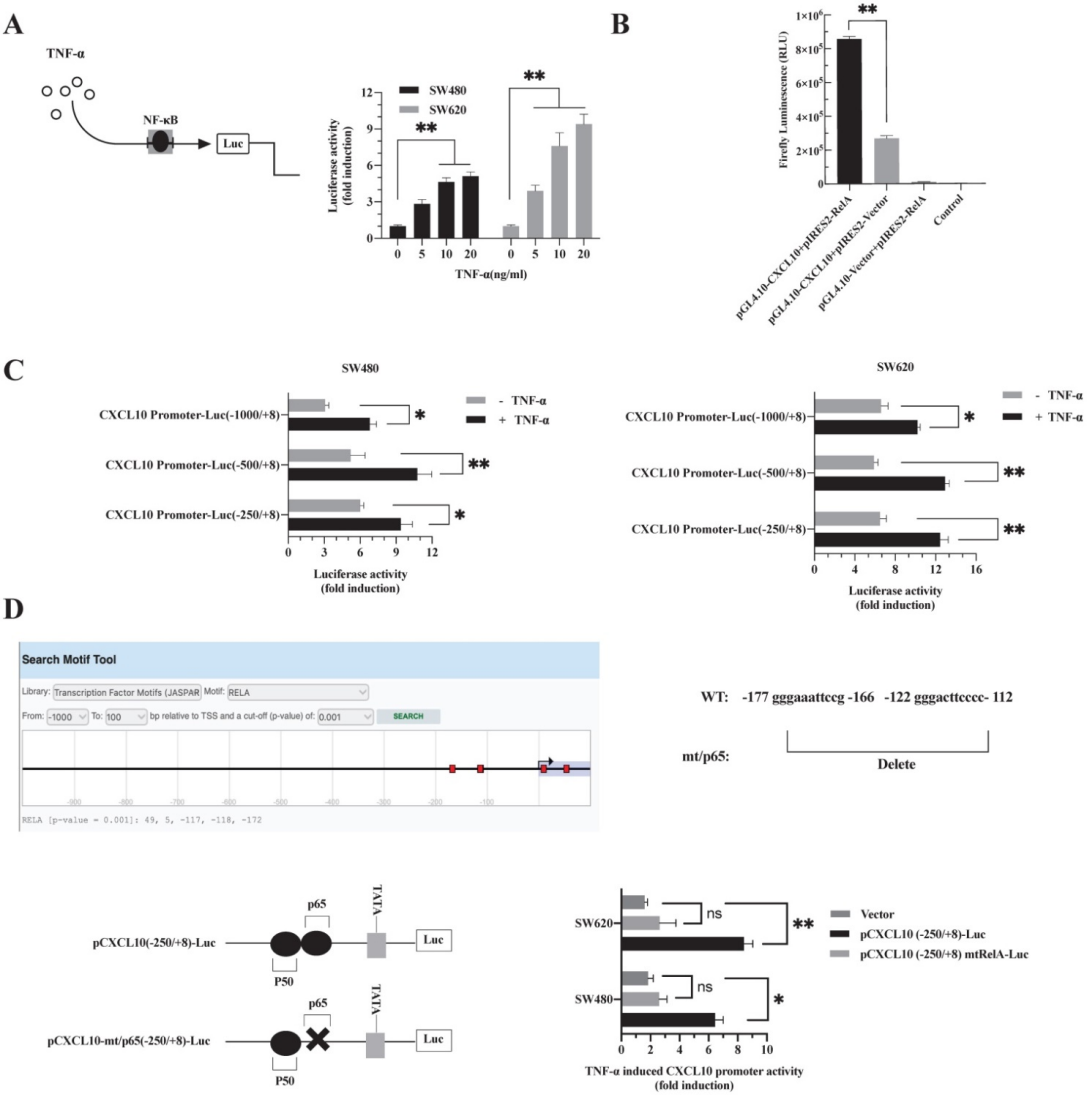
** The binding site of NF-κB p65 plays a critical role in TNF-α mediated activation of CXCL10 promoter. (A)** SW480/SW620 cells were transfected with Renilla luciferase and pNF-κB-Luc plasmids. Cells were lysed after 24 h of TNF-α (0-20 ng/ml) treatment, followed by measurement of luciferase activity. **(B)** pCXCL10-Luc (-250/+8) promoter plasmid and NF-κB-p65 (RelA) expression plasmid was transfected together or separately into SW480/SW620 cells. Cells were lysed after 24 h of TNF-α (0-20 ng/ml) treatment, followed by measurement of luciferase activity.** (C)** pCXCL10-Luc (-1000/+8), pCXCL10-Luc (-250/+8), or pCXCL10-Luc (-500/+8) promoter plasmid and NF-κB-p65 expression plasmid was transfected separately along with the Renilla luciferase plasmid into SW480/SW620 cells. Cells were lysed after 24 h of TNF-α (20 ng/ml) treatment, followed by measurement of luciferase activity.** (D)** Wild-type pCXCL10-Luc (-250/+8) or p65 binding site mutant pCXCL10 mtRelA (p65)-Luc (-250/+8) promoter plasmid was transfected separately into SW480/SW620 cells along with the Renilla luciferase plasmid. Cells were lysed after 24 h of TNF-α (20 ng/ml) treatment, followed by measurement of luciferase activity. In these experiments, all fold induction data were calculated by dividing the firefly luciferase luminescence intensity by that of Renilla luciferase. Every bar chart stands for mean ±SD (n=3). *p < 0.05, **p < 0.01, ns: not significant by the Sidak multiple comparison test.

p65 is a transcription factor in the NF-κB pathway, which is necessary to regulate various chemokine expression 50. The results are shown in Figure 2. Obviously, TNF-α stimulation promoted NF-κB pathway activation, p65 nuclear translocation may be a key signal for the final initiation of CXCL10 transcription, therefore, to confirm this inference, a luciferase reporter system with NF-κB-driven activity was constructed. In SW480/SW620 cells, TNF-α enhanced luciferase activity mediated by NF-κB depending on its dose (Figure 3A). Since the proximal part of the CXCL10 gene contains the binding site for NF-κB p65, three plasmids containing wild-type CXCL10 gene promoter fragments with different sizes were constructed. The p65 overexpression plasmid and CXCL10 promoter-reporter plasmids pCXCL10-Luc (-1000/+8), pCXCL10-Luc (-250/+8) and pCXCL10-Luc (-500/+8) were co-transfected into SW480/SW620 cells to assess luciferase reporter activity. TNF-α stimulated CXCL10 promoter activation and activated luciferase gene levels within SW480/SW620 cells (Figure 3C). Additionally, the luciferase activity of the separately transfected p65 and CXCL10 promoter plasmids was weak and was strongest when the CXCL10 cis-acting element was co-transfected with the p65 trans-acting factor (Figure 3B). This finding suggests that the binding site for NF-κB p65 may be an independent and crucial binding site in the CXCL10 promoter. To further determine whether TNF-α activates the CXCL10 promoter by targeting NF-κB, we predicted binding sites to RelA (p65) in the CXCL10 promoter via the EPD eukaryotic promoter genomics website, and the -172, -118, and -117 promotors were associated with binding to the p65. Next, the p65-related binding site in the pCXCL10-Luc promoter (-250/+8) was mutated, the wild-type and mutant plasmids were transfected into SW480/SW620 cells, respectively. According to Figure 3D, interfering with the NF-κB p65 site at the proximal promoter region of CXCL10 eliminated the TNF-α-mediated CXCL10 expression. The above findings indicate that TNF-α activates NF-κB pathway in CC cells, inducing p65 nuclear translocation and binding to CXCL10 promoter, thereby initiating CXCL10 gene transcription.

### CXCL10 promotes CC cell migration, invasion, and colony formation

**Figure 4:**
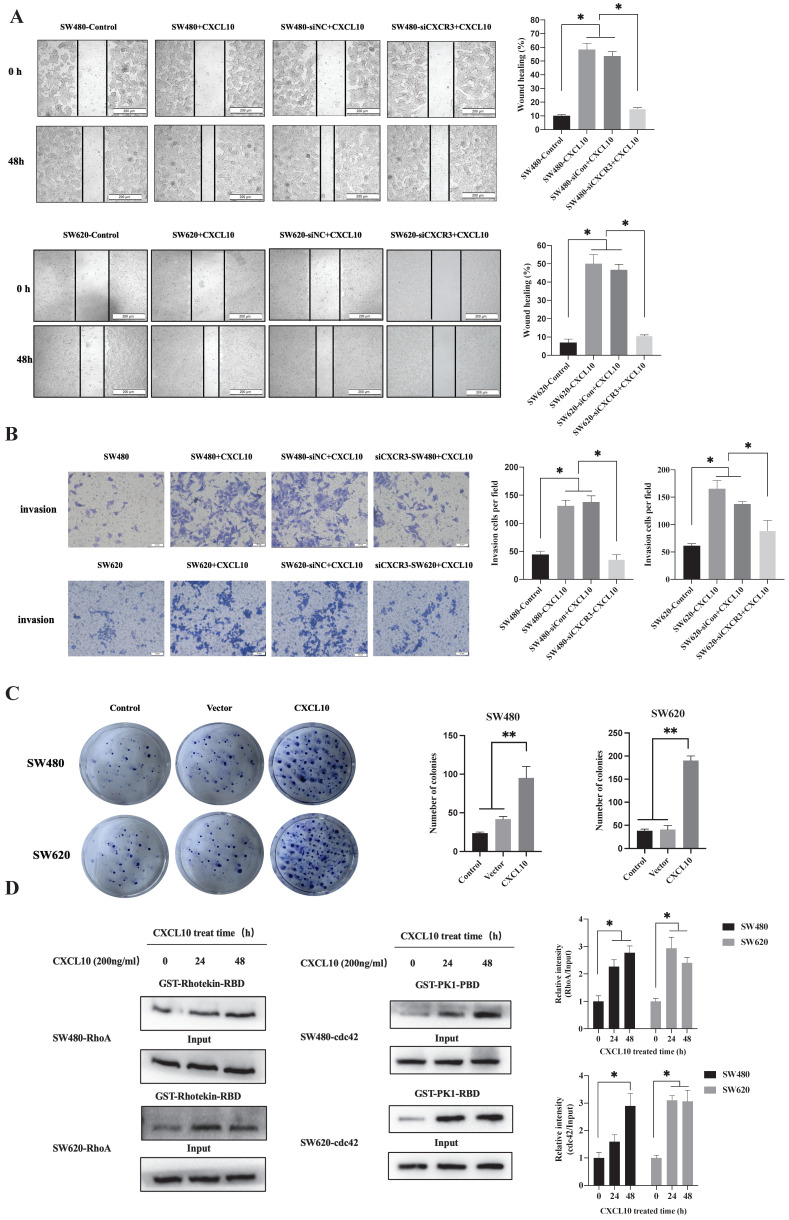
** CXCL10 regulates CC cell colony formation, invasion, and migration.** siNC or siCXCR3 was transfected into SW480/SW620 cells. Wound healing **(A)** and Transwell **(B)** assays were carried out in SW480, siNC-SW480, siCXCR3-SW480, SW620, siNC-SW620 and siCXCR3-SW620 cells after treatment with or without CXCL10 (200 ng/ml). The wound closure percentage was calculated: magnification 200×; scale bar=200 μm. Invading cell number was determined: magnification 50×; scale bar=50 μm.** (C)** Vector plasmid (Vector) or overexpressing CXCL10 plasmid (CXCL10) was transfected into SW480/SW620 cells for clone formation experiments.** (D)** CXCL10 (200 ng/ml) was used to stimulate SW480/SW620 cells to lyse total proteins at specific time points, the activated GTP-RhoA was bound to GST-Rhotekin-RBD fusion protein, and the activated GTP-cdc42 was bound to GST-PAK1-PBD fusion protein. Glutathione resin was used for the immunoprecipitation of GTP-bound cdc42 and GTP-bound RhoA. The GTP-RhoA and GTP-cdc42 activation was detected by WB assay. The band intensities of RhoA and cdc42 relative to those of the input were measured using Image J software. Every bar chart stands for mean ±SD (n=3). *p < 0.05, **p < 0.01 by Sidak multiple comparison test.

Cancer cell invasion and migration are a key biological event in tumor metastasis. However, whether CXCL10/CXCR3 axis activation after TNF-α stimulation affects biological behaviors related to CC metastasis remains unclear. First, interfering RNA knocked out CXCR3 expression within SW480/SW620 cells, WB analysis confirmed that transfection of siRNA targeting CXCR3 significantly downregulates CXCR3 levels within SW480/SW620 cells (Figure S1B). Then, wound-healing experiments revealed the role of CXCL10 in enhancing CC cell migration, but CXCR3 knockdown weakened the promotive effect of CXCL10 on CC cell migration (Figure 4A). The Transwell assays also showed that CXCL10 significantly promoted CC cell invasion across Matrigel. Similar to the results of the scratch assay, CXCR3 down-regulation reduced CXCL10's influence on CC cell invasion (Figure 4B). In addition to invasion and migration, the ability of tumor cells to grow unanchored is a necessary condition for cancer development. Colony formation experiment is one of the important experiments to analyze the self-renewal growth potential of cancer cells. As revealed by colony formation experiments, CXCL10 over-expression enhanced unanchored SW480/SW620 cell growth (Figure 4C). The activation of RhoA, Rac1/2/3 and cdc42 small GTPases exerts an important molecular switching effect on cancer cell proliferation, migration, and invasion. To investigate whether CXCL10/CXCR3 axis activation can activate the above Rho GTPases, pulldown assays based on the GST-fusion RBD/PBD were carried out. After CXCL10 stimulation, the number of RhoA bound to RBD and cdc42 bound to PBD markedly increased within SW480/SW620 cells (Figure 4D). The above findings indicate that activating the CXCL10/CXCR3 pathway promotes CC cell invasion and migration, and that among GTPases in the Rho family, cdc42 and Rho A may be involved.

### CXCL10/CXCR3 axis induced EMT in CC cells

**Figure 5:**
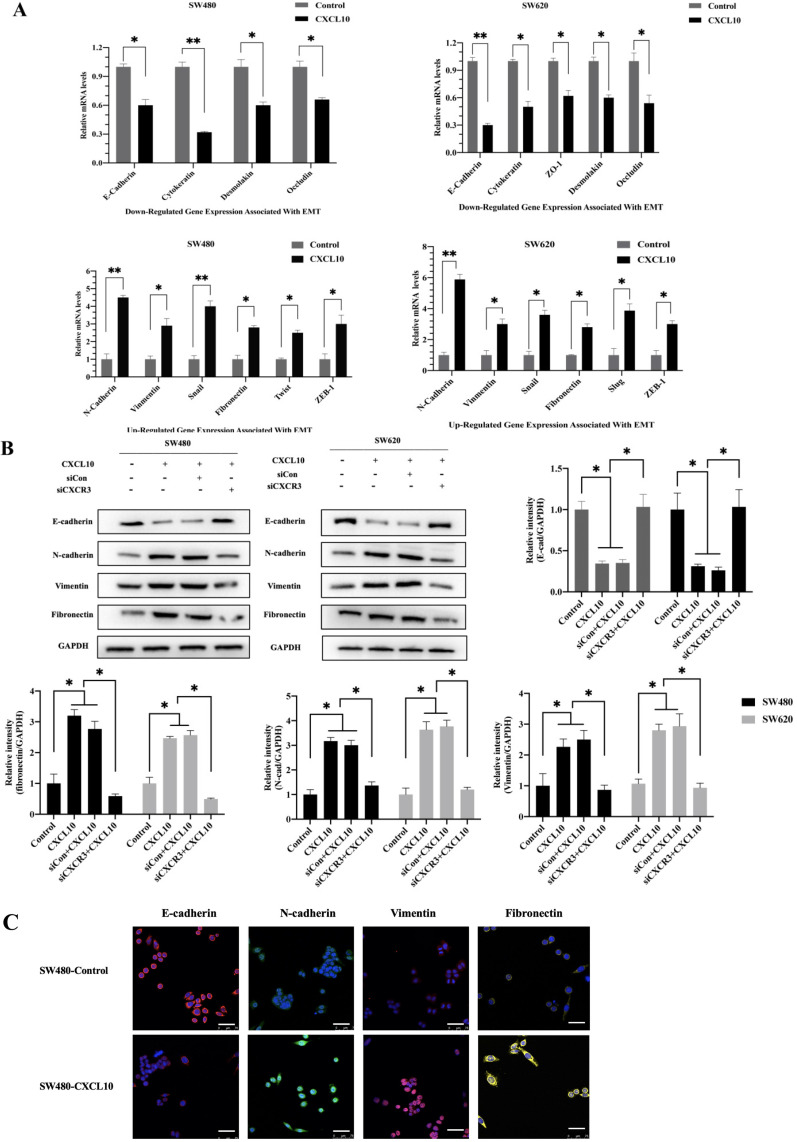

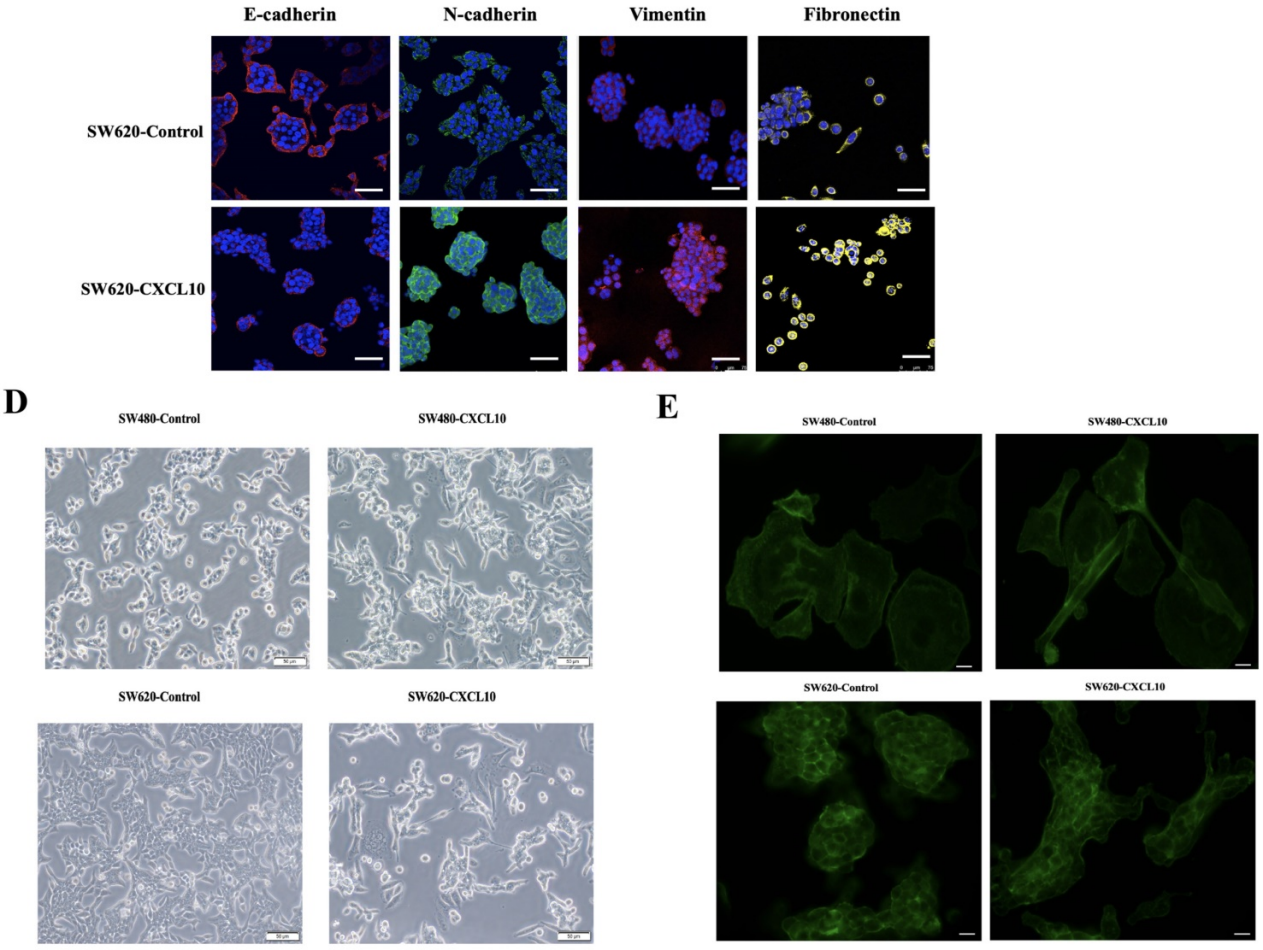
** CXCL10/CXCR3 activate EMT within CC cells. (A)** 200 ng/ml CXCL10 was used to stimulate SW480/SW620 cells for 18 h, and E-cad, Fibronectin, N-cad, Vimentin, Occludin, Cytokeratin, Desmoplakin, snail and ZO-1 expression was measured through qPCR. **(B)** siCXCR3 or siNC was transfected into SW480/SW620 cells, followed by 4 days stimulation using 200 ng/ml CXCL10, then E-cad, N-cad, Fibronectin, and Vimentin levels were measured through WB assays. **(C)** 200 ng/ml CXCL10 was used to stimulate SW480/SW620 cells for 4 days. E-cad, Vimentin, Fibronectin, and N-cad levels were measured through immunofluorescence assays, nucleus was stained blue by DAPI. Magnification 100×; the scale bars represent 75 μm. **(D)** Morphological changes within SW480/SW620 cells under 4 days of CXCL10 (200 ng/ml) treatment were examined microscopically. Magnification 50×; the scale bars represent 50 μm. **(E)** SW480/SW620 cells treated with CXCL10 (200 ng/ml) for 4 days, F-actin was stained Green with Actin-Tracker Green, and the actin reorganization was analyzed. Magnification 100×; the scale bars represent 20 μm. Every bar chart stands for mean ±SD (n=3). *p < 0.05, **p < 0.01 by Sidak multiple comparison test.

EMT represents a vital event during tumor development, which has a great influence on tumor migration and invasiveness. Therefore, to investigate the effect of CXCL10/CXCR3 axis activation on EMT in CC cells, qPCR, WB analysis and immunofluorescence staining were used for assessing mesenchymal and epithelial marker levels in CC cells after CXCL10 stimulation. According to qPCR assay, diverse EMT-related gene levels were changed in CC cells after CXCL10 stimulation; specifically, the epithelial marker gene mRNA levels (E-cad, ZO-1, Occludin and Cytokeratin) decreased, and the mesenchymal marker mRNA levels (Vimentin, N-cad, and Fibronectin) increased (Figure 5A). Similarly, CXCL10 significantly increased the mesenchymal marker protein levels (N-cadherin, Vimentin and Fibronectin) within SW480/SW620 cells and reduced epithelial marker protein level (E-cadherin), and CXCR3 knockdown in these cell lines significantly reduced the influence of CXCL10 on EMT-related genes (Figure 5B). Similar results were observed with immunofluorescence staining (Figure 5C). In addition, CXCL10 treatment altered the morphology of CC cells, which gained the spindle-like fibroblastic phenotype from the cobblestone-like epithelial phenotype, and the cell spacing increased (Figure 5D). Further, actin microfilament staining showed that after CXCL10 stimulation, the spindle-like morphology of the CC cells was changed and F-actin polymerization at the cell edge was increased, which may indicate enhancement of cell invasion and migration (Figure 5E). Collectively, the above outcomes suggest that CXCL10/CXCR3 axis activation promotes EMT in CC cells.

### CXCL10 induced EMT via PI3K/Akt/GSK-3β/Snail pathway within CC

**Figure 6:**
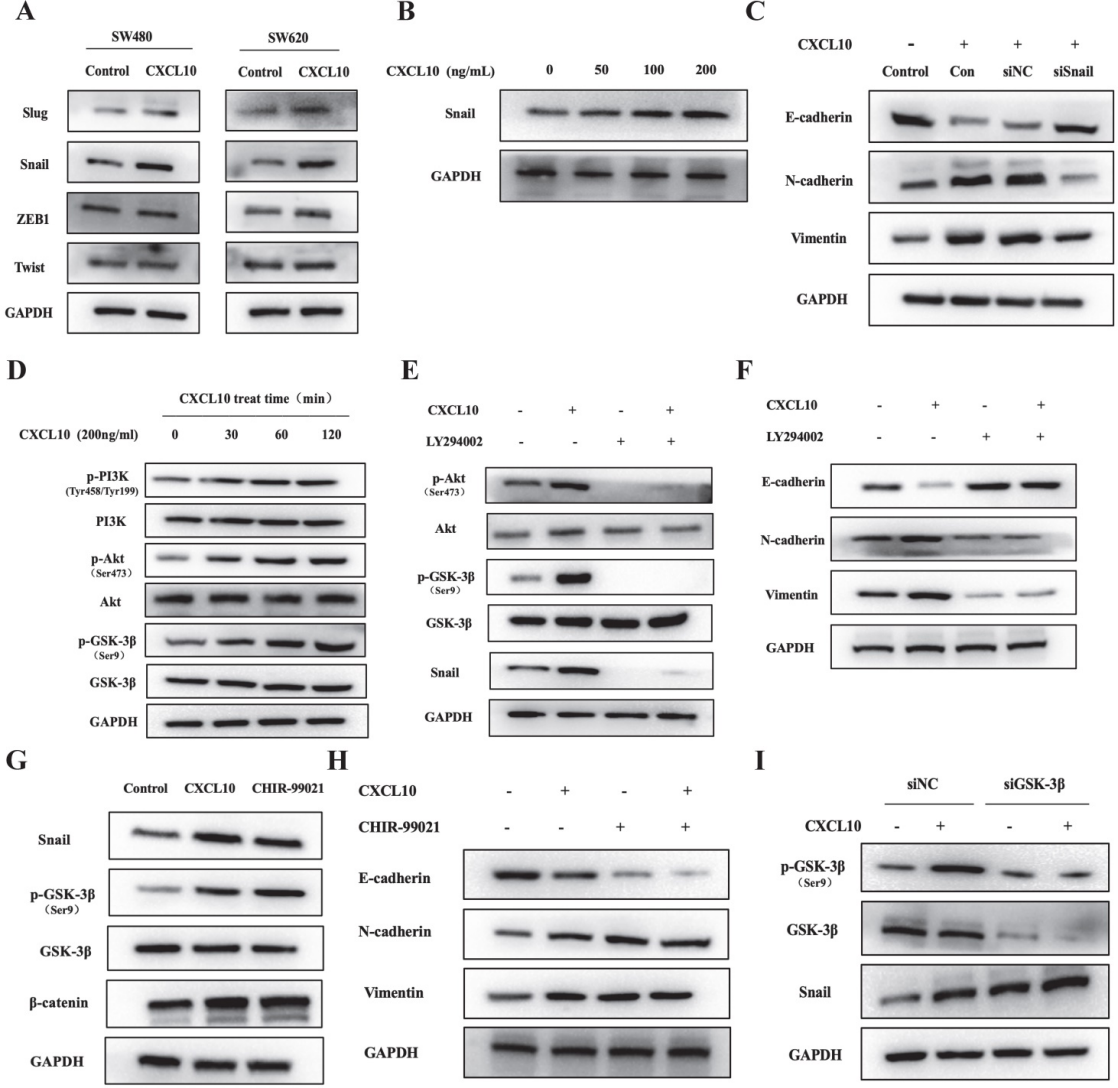
** CXCL10 regulates EMT in CC cells through the PI3K/Akt/GSK-3β/Snail pathway. (A)** CXCL10 (200 ng/ml) was used to stimulate SW480/SW620 cells for 4 days, and protein levels of Slug, Snail, ZEB1 and Twist were measured through WB assays. **(B)** CXCL10 (0-200 ng/ml) was used to treat SW620 cells for 4 days, then Snail protein level was examined through WB assays. **(C)** siSnail or siNC was transfected into SW620 cells, followed by 4 days of stimulation with CXCL10 (200 ng/ml). E-cad, N-cad and Vimentin protein expression was evaluated through WB assays. **(D)** CXCL10 (200 ng/ml) was used to stimulate SW620 cells at specified time point. p-Akt/Akt, p-PI3K/PI3K and p-GSK-3β/GSK-3β levels were detected by WB assay. **(E-F)** LY294002 (10 mM) was used to treat SW620 cells for 1 h before CXCL10 (200 ng/ml) stimulation, the Snail, Vimentin, N-cad, E-cad, p-Akt/Akt, and p-GSK-3β/GSK-3β expression was measured through WB assays. **(G-H)** SW620 cells were pretreated with CHIR-99021 (40 mM) for 1 h prior to CXCL10 (200 ng/ml) stimulation, the Snail, p-GSK-3β/GSK-3β, β-catenin, Vimentin, N-cad, and E-cad protein expression was measured through WB assay. **(I)** siGSK-3β or siNC was transfected into SW620 cells, followed by 24 h of CXCL10 (200 ng/ml) stimulation. Snail and p-GSK-3β/GSK-3β protein expression was analyzed by WB assays.

During CXCL10-induced EMT of CC cells, Snail, ZEB1, Twist and Slug mRNA levels were changed (Figure 5A). Interestingly, the WB analysis results showed that CXCL10/CXCR3 axis activation markedly enhanced the protein expression of the only Snail, but made no difference to ZEB1, Twist, and Slug protein levels (Figures 6A, S3A). Moreover, the higher the CXCL10 stimulation concentration was, the greater the upregulation of Snail protein expression in colon cancer cells (Figures 6B, S3B). However, after knockdown of Snail, CXCL10/CXCR3 axis activation did not lead to the same changes in EMT marker levels within CC cells described above (Figures 6C, S3C). Based on the above findings, we can conclude that Snail upregulation participates in the CXCL10/CXCR3 axis activation induced EMT within CC cells. Activating PI3K/Akt pathway has been reported to suppress GSK-3β phosphorylation; besides, as GSK-3β is the main regulator of Snail, its inactivation can lead to upregulation of Snail expression 51, 52. Does CXCL10/CXCR3 axis activation also upregulate Snail expression in CC cells through the above pathway? To answer this question, WB assay was performed to analyze PI3K/Akt/GSK-3β pathway activation within CC cells after CXCL10 stimulation. As shown in Figures 6D and S3D, PI3K, Akt and GSK-3β in CC cells were significantly phosphorylated at different durations of CXCL10 stimulation. However, after CC cells were treated with a PI3K inhibitor, CXCL10 stimulation did not cause phosphorylation of PI3K, GSK-3β and Akt; moreover, Snail expression was significantly decreased (Figures 6E, S3E). More importantly, after PI3K was inhibited, CXCL10 stimulation did not downregulate E-cadherin level or upregulate Vimentin and N-cadherin levels (Figures 6F, S3F). Besides, after GSK-3β inhibitor treatment, not only GSK-3β phosphorylation but also GSK-3β regulated Snail and β-catenin were upregulated in these cells, consistent with the alterations of their levels within CC cells after CXCL10 stimulation (Figures 6G, S3G). Moreover, when GSK-3β was suppressed, Vimentin and N-cadherin levels increased, while E-cadherin level was reduced in CC cells regardless of CXCL10 stimulation or not (Figures 6H, S3H). In addition, when the expression of GSK-3β in CC cells decreased by siRNA, Snail expression increased, which was not affected by CXCL10 stimulation or not (Figures 6I, S3I), these results further confirmed the regulatory effect of GSK-3β on Snail expression in CC cells. The above results indicate that CXCL10/CXCR3 axis activation can in turn activate PI3K/Akt, thus suppressing GSK-3β phosphorylation and subsequently up-regulating Snail expression, thereby regulating EMT in CC cells.

### CXCL10 increases tumor growth and CC cell metastases *in vivo*

**Figure 7:**
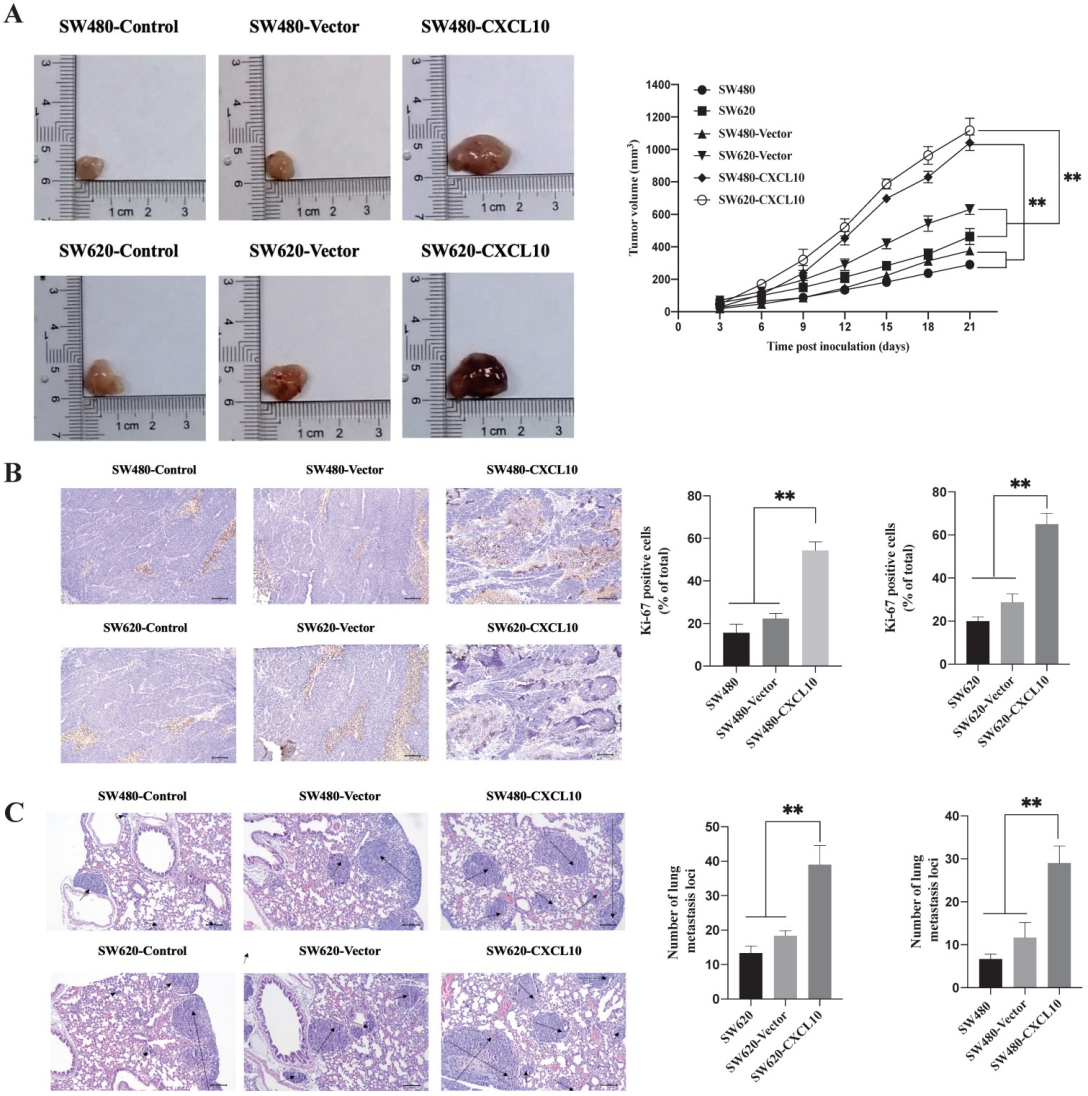
** CXCL10 increases tumor growth and CC cell metastasis *in vivo* (A)** Typical images showing tumor masses collected in nude mice transplanted with SW480, SW620, SW480-Vector, SW620-Vector, SW480-CXCL10, SW620-CXCL10 cells. Tumor length and width were detected using a ruler per 3 days, then tumor size was determined (length^2^×width^2^×0.5). **(B)** The cell proliferation marker Ki-67 within CC was measured through immunohistochemistry, the Ki-67-positive cell proportion was calculated. the scale bars represent 100 μm. **(C)** The lung metastatic lesion number was used to analyze and calculate by H&E staining. (Black arrows indicate metastatic sites). the scale bars represent 100 μm. Every bar chart stands for mean ±SD (n=3). **p < 0.01 by Sidak multiple comparison test.

The above results indicate that CXCL10/CXCR3 axis activation can promote the metastasis-related biotic activities of CC cells *in vitro*. To explore whether these effects exist *in vivo*, SW480 and SW620 cell lines stably overexpressing CXCL10 (SW480-CXCL10 and SW620-CXCL10) and the corresponding control cell lines (SW480-Vector and SW620-Vector) were generated. The generated cell lines and the wild-type SW480/SW620 cell lines were subcutaneously inoculated into nude mice for evaluation of tumorigenesis. As shown in Figure 7A, the proliferation rate of CC cells overexpressing CXCL10 *in vivo* was markedly enhanced compared with corresponding controls. Unsurprisingly, Ki-67 expression within the tissues of tumors formed by CXCL10-overexpressing CC cells remarkably increased compared with those formed by related control and wild-type CC cells (Figure 7B). In addition, mice were given injection of the above-mentioned CC cells via the tail vein for observing formation of lung metastases. Compared with the corresponding control and wild-type CC cells, SW480-CXCL10 and SW620-CXCL10 cells formed more tumor metastases in the lungs of nude mice, indicating that upregulation of CXCL10 expression increased the incidence of lung metastases (Figure 7C). Based on these results, CXCL10/CXCR3 pathway activation enhances CC cell metastasis *in vivo* and that CXCL10 expression can significantly enhance the tumorigenicity and mobility and promote the growth of CC cells, as well as their migration to distant organs, *in vivo*.

## Discussion

One of the main challenges of cancer treatment is metastases caused by chronic inflammation. Cancer-related inflammation promotes cancerous cell growth, angiogenesis and survival, and its proposed mechanism, though, has yet to be completely elucidated. Inflammatory mediators such as TGF-β and IL-6 have been reported to aid the metastasis and invasion of cancer cells 53, TNF-α mediates the transition from chronic inflammation to cancer by acting as a master switch. TNF-α has a broad variety of biological functions as a significant proinflammatory cytokine, including promoting inflammation, apoptosis, cell proliferation, and differentiation 38, 46, 47. Even though TNF-α is considered an anticancer drug, there is evidence that long-term increases in TNF-α levels can facilitate tumor formation, invasion, and metastases 38, 47, 54, 55. TNF-α is formed by tumor-associated stromal cells within TME and is involved in diverse chemokine expression as well as the modulation of tumor invasion and metastases. In addition, tumors have been found to have dysregulated expression of chemokine family genes. Some researchers believe that CXC chemokines and receptors promote the metastasis of tumors 56, 57. CXCL10 is produced by many tumor cells and is found in high concentrations in a few human diseases and cancers. We hypothesized that the mobilization of Th1 cells and the upregulation of TNF-α in the inflammatory tumour microenvironment could be the cause of this up regulation. In exchange, TNF-α promote the expression of CXCL10, resulting in CXCL10 and Th1 responses that are amplified by positive feedback. Understanding the control of CXCL10, which is linked to disease incidence, aids in the development of therapeutic action strategies 58. This study first discovered the role of TNF-α in inducing CXCL10 up-regulation in CC cells, in this research. TNF-α triggered PI3K/Akt and p38 MAPK parallel signal transduction pathways while stimulating the downstream NF-κB pathway p65 into the nucleus to initiate CXCL10 transcription by phosphorylation, as supported by WB analysis and qPCR experiments. These findings indicate that the NF-κB p65 binding site might be an isolated and critical binding site within CXCL10 promoter.

p65 is suggested to form heterodimer or homologous complex with NF-κB family members 59-61. The dimers bind to the target DNA molecules' NF-κB binding sites. Due to the differences in molecular specificity and affinity of different genes, the degree of binding of dimer to NF-κB targets is different. Members of the NF-κB family control the accessibility of several promoters to transcription factors, thus controlling gene expression indirectly. As a result, NF-κB serves as the multifunctional transcription factor that is discovered in nearly each cell type, which regulates a variety of signal transduction pathways, such as pathways governing apoptosis, differentiation, immunity, inflammation, and cell proliferation. We investigated the interaction between CXCL10 and the NF-κB pathway because of this finding. Overexpression of p65 was effectively incorporated with expression of the CXCL10 promoter in our luciferase reporter experiment, demonstrating that TNF-α induced CXCL10 transcription in SW480/SW620 cells required the binding of CXCL10 promoter to p65.

Moreover, CXCR3, the receptor for CXCL10, is found to show over-expression within many malignant tumors, which has tumor growth-promoting activity and is associated with dismal survival and metastasis of many cancers 29,62-64. Goldberg-Bittman reported that CXCR3 was overexpressed in breast cancer 65. According to Hu, CXCR3 over-expression was detected within gastric cancer (GC), while it was expressed at reduced levels in nontumor tissues 66. To explore more about the role of CXCR3 in TNF-α-induced CC metastases, researchers used qPCR, WB analysis, and flow cytometry to assess CXCR3 expression in CC cells with and without TNF-α stimulation, the results showed that CXCR3 showed over-expression within CC cells, with its expression being regulated by TNF-α. In addition, CXCR3 expression increased within SW620 metastatic CC cells than in SW480 primary CC cells. The chemokine-receptor interactions have been thought to influence cancer metastasis. New evidence shows that the CXCR3, CXCL9-CXCR11 signaling networks impact tumor cell metastasis and proliferation, which is a factor indicating poor prognosis 31, 62, 67. The binding of CXCL10 with its receptor CXCR3 has been reported to enhance lymph node metastasis and tumorigenesis in human glioma, CC, and melanoma 27, 29, 68. In addition, in a mouse model, a small molecule CXCR3 antagonist blocked lung metastasis of breast cancer 28. We confirmed that CXCL10 can enhance CXCR3-expressing CC cell invasion and migration via cell wound healing assays and Transwell experiments and showed that interference with CXCR3 expression weakens the promotion of CXCL10 on CC cell invasion and migration. Additionally, we inoculated CC cells overexpressing CXCL10 into nude mice subcutaneously and confirmed that CXCL10 promoted tumor growth in mice by observing tumor volume changes and detecting Ki-67 expression by immunohistochemistry. Here, genes closely related to cell migration and invasiveness were also analyzed by WB analysis and qPCR. The promotion of CXCL10 on CC cell migration and invasion was accompanied by upregulated expression of RhoA, cdc42 and other genes. Activation of RhoA can led to increased intracellular oxidative stress; regulated intercellular adhesion mediated by cadherins; increased the expression of fibrogenic growth factors, extracellular matrix components and inflammatory cytokines; and changed tumor cells' invasion and migration. It suggests that the invasion of CC cells may be accompanied by a high probability of EMT 69.

**Figure 8:**
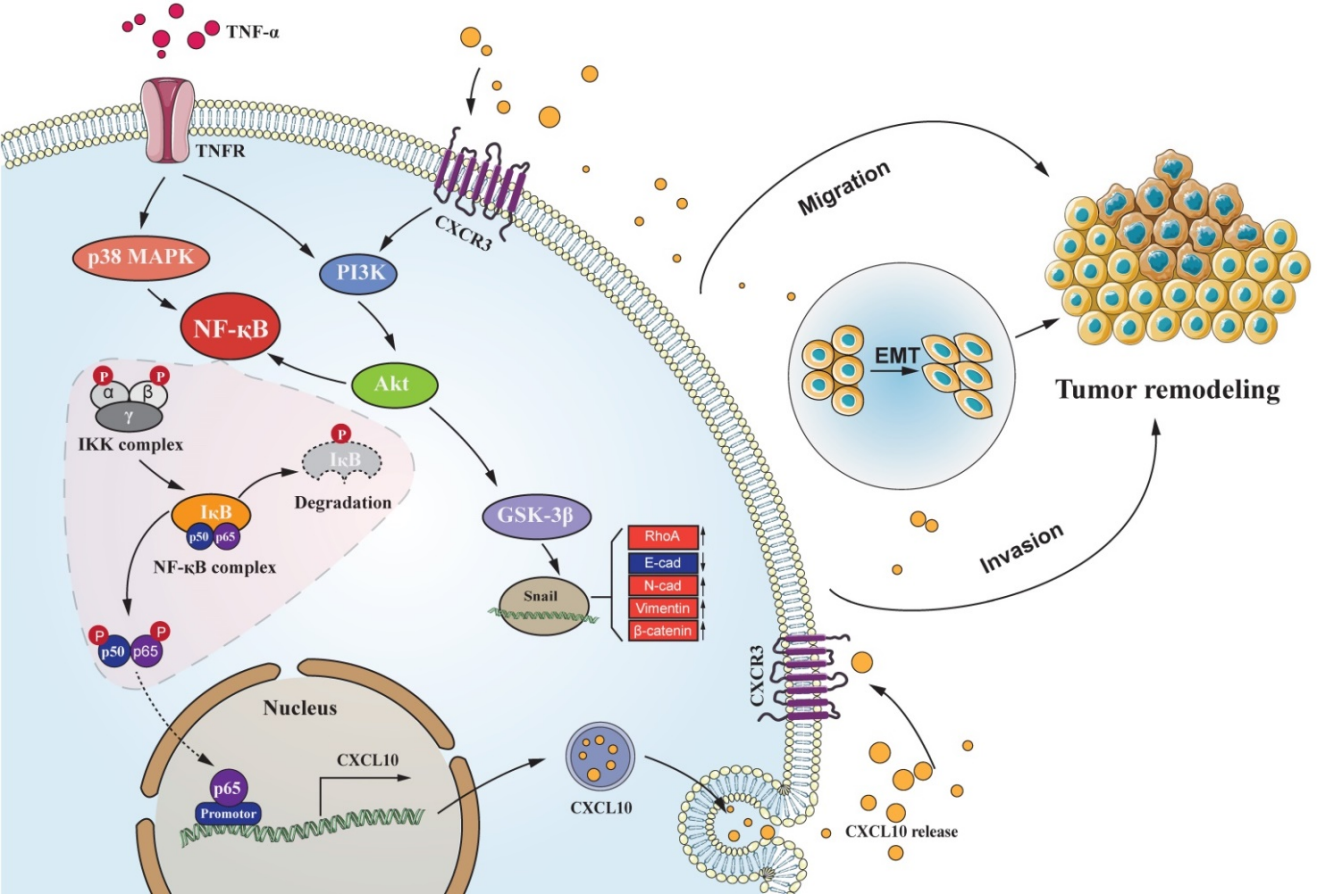
** Schematic of the signaling pathways involved in TNF-α augments CXCL10/CXCR3 axis to promote CC cell EMT, invasion and migration.** TNF-α up-regulates CXCL10 level within CC through p38 MAPK, NF-κB and PI3K/Akt pathways. Besides, CXCL10 promotes migration and invasion of CXCR3-expressing autogenous CC cells; on the other hand, CXCL10/CXCR3 regulates EMT in CC cells via PI3K/Akt/GSK-3β/Snail pathway. All of these changes in cell biological activity can promote colon cancer metastasis.

CC follows a developmental process of progression from normal mucosa to polyp to adenoma to carcinoma. EMT represents an important process during tumor evolution, which plays a significant part in metastases of tumor cells. The essential characteristics of EMT progression are the decreased E-cadherin level but increased N-cadherin level to regulate the adhesion between tumor cells; thus, enhancing the invasiveness of cancer cells. For exploring the correlation of CXCL10 with EMT within CC, we used SW480 primary CC cells and SW620 metastatic CC cells, which were derived from the same patient. However, SW620 cell exhibits higher invasiveness, to jointly verify the effect of CXCL10/CXCR3 on EMT in CC cells. After CXCL10 treatment in SW480/SW620 cells, vimentin and N-cadherin expression increased, whereas E-cadherin expression declined. After siRNA-mediated interference with CXCR3 level within the above cells, CXCL10 at identical concentration did not cause EMT within SW480/SW620 cells. The PI3K/Akt pathway has effect on invasion and migration 70, the expression of CXCL10/CXCR3 has been shown to increase PI3K/Akt activity in human perivascular cells 71. However, whether PI3K/Akt is activated by CXCL10/CXCR3 in CC cells is unclear. The researchers demonstrated that activation of the CXCL10/CXCR3 axis induces PI3K/Akt pathway activation and EMT, further treatment with an Akt inhibitor showed that inhibition of this pathway could also abolish CXCL10-mediated EMT. Based on these results, PI3K/Akt pathway plays a necessary role in CXCL10 activation mediated EMT within CC cells. Previous studies confirmed that many other transcription factors, including ZEB1, Twist and Slug, regulate EMT during cancer progression 72, 73. We further examined these transcription factors mRNA and protein expression within SW620 cells after CXCL10 stimulation. Snail mRNA and protein expression only changed significantly under CXCL10 treatment; in addition, interference with Snail expression also increased E-cadherin expression within SW620 cells, affecting EMT development. Based on these findings, we can conclude that Snail activates EMT within CXCL10-stimulated CC cells. Snail, the zinc finger protein, has been considered to be the important link to EMT occurrence 74, and abnormal Snail level was markedly related to lymph node metastasis (LNM) in CC 75. Snail, on the other hand, represents the extremely unstable protein that has a short half-life, and its transcriptional and posttranscriptional levels are modulated by a complicated signal transduction network 76. GSK-3β is considered to be a major kinase responsible for Snail localization and protein stability 77, 78. In this study, we confirmed through pretreatment with related pathway inhibitors and siRNA interference experiments that activation of Akt signal inhibited GSK-3β activity and weakened correlation of Snail with GSK-3β. Weakening of this interaction increased the stability of Snail; in turn, translocation of the stable Snail protein into the nucleus was promoted, subsequently reducing E-cadherin level but increasing vimentin and N-cadherin levels, and finally inducing EMT and promoting tumor metastases. Based on these results, we confirm that TNF-α as the key inflammation regulator within TME, and TNF-α upregulates CXCL10 through the NF-κB, PI3K/Akt, and p38 MAPK pathways, acts on cancer cells with high expression of CXCR3, promotes CC migration and invasiveness, and induces EMT in CC by PI3K/Akt/GSK-3β/Snail pathway. This mechanism is visually depicted in the graphical abstract (Figure 8). On the basis of these reports, we may also propose a theoretical model for the mechanism by which tumor cells achieve organ-specific metastases based on chemokines, as follows. Tumor cells highly express chemokine receptors, while target organs highly express chemokine ligands. Tumor cells ultimately achieve specific metastasis to these organs via the specific binding ability of chemokines to their receptors. Besides, cancer cells also produce chemokines, which bind to chemokine receptors on their own cell membrane and then activate the corresponding downstream signaling pathway and promote EMT of these tumor cells, thus significantly enhancing cell invasion and migration. In summary, these findings improve our understanding of the metastatic mechanism and function of CXCL10/CXCR3 in CC, provide more insight into the signal transduction underlying CC metastases, and may augment the arsenal of treatment options for CC.

## Conclusions

Our study confirmed that TNF-α upregulates the CXCL10 expression within CC cells via the p38 MAPK, NF-κB and PI3K/Akt pathways. The enhanced CXCL10/CXCR3 axis activity promotes CC cell migration and invasiveness and induces CC cell EMT by PI3K/Akt/GSK-3β/Snail pathway. These signaling pathways provide better and comprehensive mechanism for CC metastases exploration and can be able to provide a groundbreaking field for CC treatment approaches.

## Supplementary Material

Supplementary figures.

## Abbreviations

CC: colon cancer
CXCR3: chemokine C-X-C Motif receptor 3
CXCL10: CXC chemokine C-X-C Motif ligand 10
TNF-α: tumor necrosis factor-alpha
GEPIA: Gene Expression Profiling Interactive Analysis
PI3K: phosphatidylinositol 3-kinase
IKK: IκB kinase
Akt: protein kinase B
MAPK: mitogen-activated protein kinase
IκB: Inhibitor of κB
GSK-3β: Glycogen synthase kinase-3β
NF-κB: Nuclear factor kappa-B paired box gene
EMT: Epithelial-mesenchymal transition
cdc42: cell division cycle 42
RhoA: ras homolog family member A
siNC: small, interfering non-specific control
