# Supplementary material for: TNF-α augments CXCL10/CXCR3 axis activity to induce Epithelial-Mesenchymal Transition in colon cancer cell
Source: Int J Biol Sci. 2021 Jun 26;17(11):2683–702. doi: 10.7150/ijbs.61350 (PMC8326125; doi:10.7150/ijbs.61350)
Supplement: Supplementary file 1 — Supplementary figures. [file ijbsv17p2683s1.pdf]

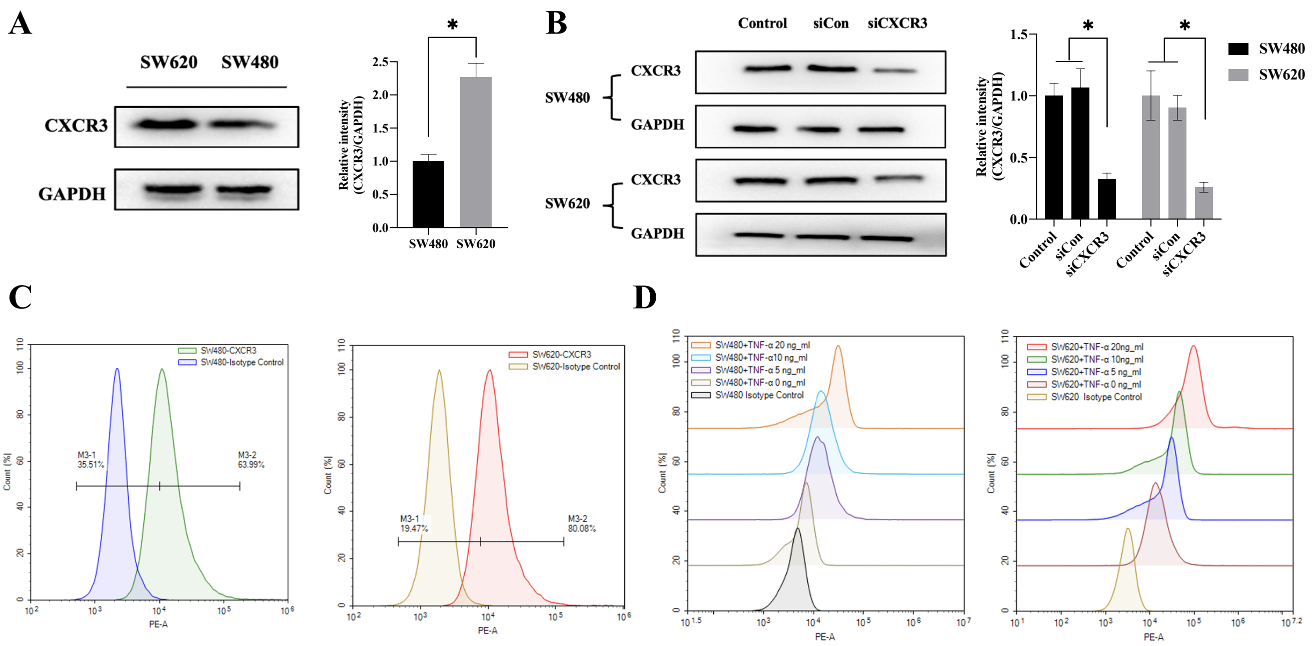

FigureS1. TNF-α regulates the expression of CXCR3 and the construction of siCXCR3 cells in SW480/SW620 (A)SW480/SW620 cultured in 6-well plates for 24h, CXCR3 protein expression was measured by WB. The band intensities of CXCR3 relative to GAPDH level were measured using ImageJ software. Each bar represents the mean ± SD of three independent experiments. *$P < 0.05$ by Student's t-test. **(B)**SW480/SW620 cells were transfected with the control siNC or siCXCR3 and the expression of CXCR3 was determined by the Western blot assay. The band intensities of CXCR3 relative to GAPDH level were measured using ImageJ software. each bar represents the mean ± SD of three independent experiments. *$P < 0.05$, **$P < 0.01$ by Sidak'smultiple comparisons. **(C)**SW480/SW620 cultured in 6-well plates for 24h, CXCR3 protein expression on the membrane was measured by flow cytometry. **(D)**SW480/SW620 cultured in 6-well plates were incubated with different concentrations of TNF-α (0-20ng/ml). After 24 h, CXCR3 expression on the membrane was measured by flow cytometry.

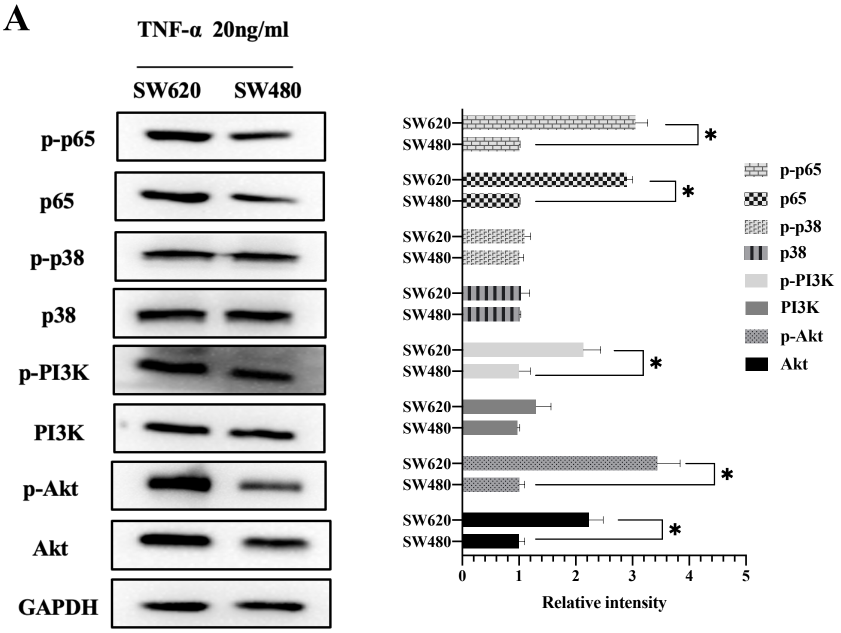

FigureS2. Comparison of TNF-α induced activation of p38 MAPK，PI3K/Akt and NF-κB pathways in SW480/SW620 cells(A) SW620/SW480 cells were stimulated with 20ng/mL TNF-α for 2h. The blots were probed with antibodies against p-p65/p65, p-p38/p38, p-PI3K/PI3K, and p-Akt/Akt. An antibody against GAPDH was used to reprove the blots for normalization, the band intensities of p-p38/p38, p-PI3K/PI3K, p-Akt/Akt and p-p65/p65 relative to GAPDH level were measured using ImageJ software. each bar represents the mean ± SD of three independent experiments. *$P < 0.05$ by Student's t-test.

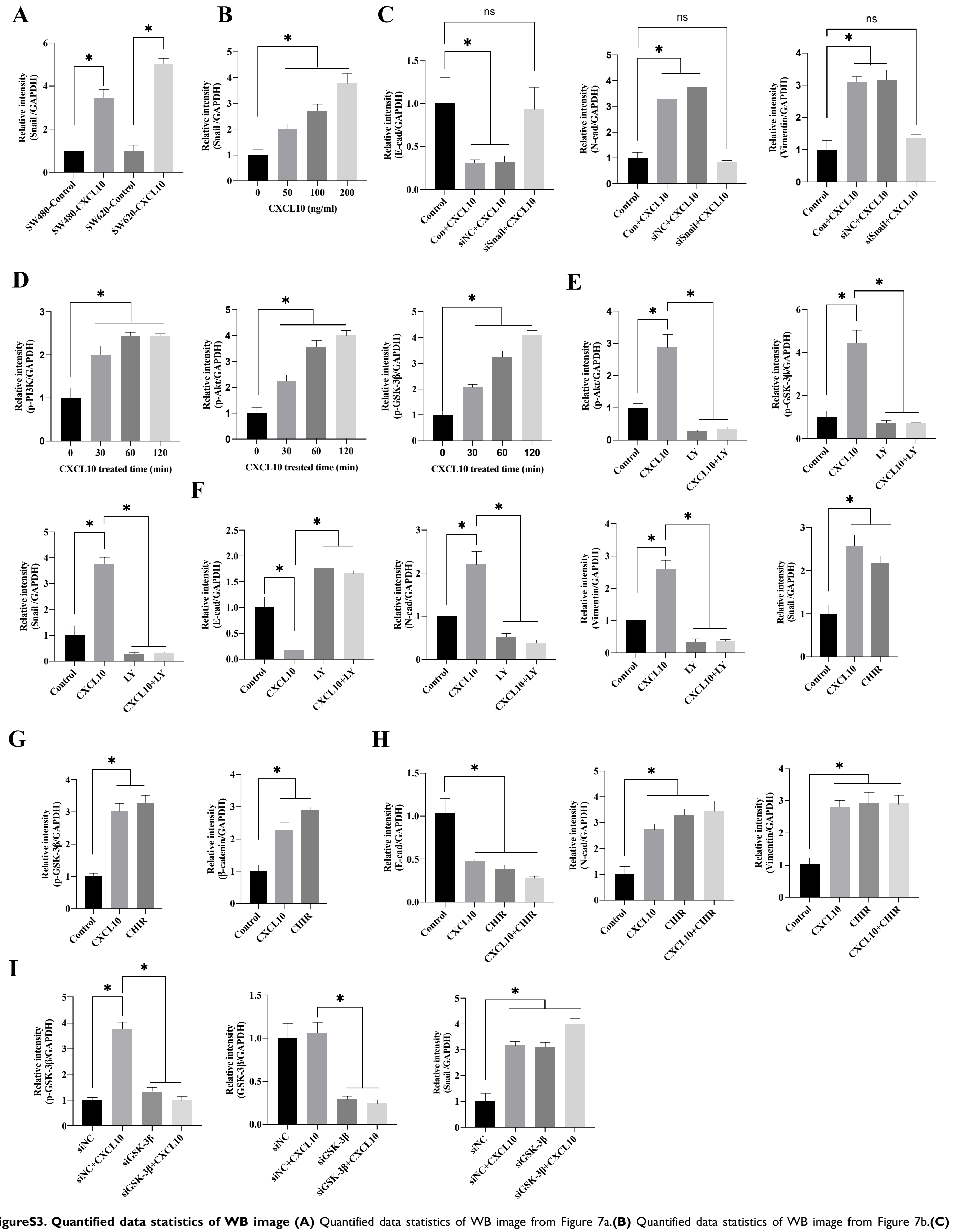

FigureS3. Quantified data statistics of WB image (A) Quantified data statistics of WB image from Figure 7a.**(B)** Quantified data statistics of WB image from Figure 7b.**(C)** Quantified data statistics of WB image from Figure 7c.**(D)** Quantified data statistics of WB image from Figure 7d.**(E)**Quantified data statistics of WB image from Figure 7e.**(F)** Quantified data statistics of WB image from Figure 7f.**(G)** Quantified data statistics of WB image from Figure 7g.**(H)** Quantified data statistics of WB image from Figure 7h.**(I)** Quantified data statistics of WB image from Figure 7i. each bar represents the mean ± SD of three independent experiments. *$P < 0.05$ NS: Not Significant by Sidak'smultiple comparisons.
